# Associations between Demographic Characteristics, Lifestyle Factors and School-Related Conditions and Symptoms of Mental Health Problems in Norwegian Upper Secondary School Students

**DOI:** 10.3390/ijerph19159575

**Published:** 2022-08-04

**Authors:** Svein Barene, Andreas Ruud-Tronsmoen, Patrick Foss Johansen

**Affiliations:** Department of Public Health and Sport Sciences, Inland Norway University of Applied Sciences, 2418 Elverum, Norway

**Keywords:** lifestyle factors, school well-being, physical education, mental health, high school students

## Abstract

Background: The purpose of this study was to evaluate the associations between demographic characteristics, lifestyle factors and school-related conditions, and symptoms of mental health problems in Norwegian upper secondary school students following the COVID-19 pandemic. Methods: In this cross-sectional study design we used a binary logistic regression model to evaluate potential associations between the predictors and dependent variable. Results: The following six predictors had a statistically significant impact on symptoms of mental health problems; gender effect of being a girl (*p* < 0.001), self-perceived body image (*p* < 0.001), sleep problems (*p* < 0.001), dietary habits (*p* = 0.033), school satisfaction (*p* = 0.013), and satisfaction with physical education (PE) class participation (*p* = 0.025). Conclusions: Being a girl was associated with a 315% higher probability of reporting symptoms of mental health problems than boys, whereas one unit increase in sleep problems showed a 192% higher probability of symptoms of mental health problems. Furthermore, a one unit increase on the respective beneficial predictors’ scales was associated with the following percentage having a lower probability of reporting symptoms of mental health problems; self-perceived body image (59%), dietary habits (58%), school satisfaction (82%), and satisfaction with PE class participation (68%).

## 1. Introduction

A sound mental health and well-being are crucial for the individual’s perceived quality of life, as well as for a population’s health in general [1,2]. Although they are often not detected until adulthood, most mental problems occur in early adolescence [3], making this a crucial period for the development of positive mental health [4]. According to recent estimates, approximately 14% of 10–19-year-olds experience a mental health problem worldwide [5]. Based on the ever-increasing incidence worldwide [6], with a worrying deterioration among adolescents in middle- and high-income countries during recent decades [7] that has been further exacerbated in recent years due to the COVID-19 pandemic [8,9], improving people’s mental health and wellbeing has been identified as an important public health goal [10].

The most common mental health problems among adolescents are generalized anxiety and depression [11,12]. The consequences of depression and anxiety at a young age can impair their development through lower educational attainment, result in dropping out of school, impair their social relationships, and increase the risk of substance abuse, mental health problems, and suicide [12].

Previous research has identified various factors with relevance for mental health problems in adolescents, including gender [13], socioeconomic inequalities [14], being obese [15], academic performance and academic pressure [16], physical activity level [17,18], social media use [12], dietary habits [19,20], and substance use [21]. However, the effects of the proposed preventive measures related to these various factors can be considered relatively modest given the ever-increasing incidence of mental health problems among adolescents.

School satisfaction refers to a student’s subjective cognitive appraisal of the quality of his or her school life and can be linked to the construct of quality of life [22]. Given that adolescents spend a large amount of time in school, school is considered a suitable arena to promote adolescent mental health and wellbeing [10,23]. Furthermore, physical education (PE) in school is recognized as a main tool for health promotion, and a focus on the students’ subjective experiences rather than on improving skill mastery and physical fitness is recommended for an internalization of physical activity behaviours associated with lifelong health [24]. In accordance with the Norwegian Education Act § 9A-2, all students are entitled to a good physical and psychosocial environment conductive to health, wellbeing and learning [25]. With the aim of promoting sound physical and mental health among students, as well as giving them opportunity to make responsible life choices, the Norwegian Directorate for Education and Training in 2020 also introduced the interdisciplinary topic ‘Public health and life skills’ in the new National K-12 curriculum [26], but effects of this measure are still poorly documented.

The purpose of this study was to evaluate associations between demographic characteristics, lifestyle factors, and school-related conditions, respectively, and symptoms of mental health problems in Norwegian upper secondary school students. In this connection, we would like to put forward the following hypotheses that we wish to examine; (1) Girls experience symptoms of mental health problems to a greater extent than boys, (2) Frequent contact with friends is associated with less symptoms of mental health problems, (3) Students with a positive body image have a lower risk of reporting mental health problems than those who report body image dissatisfaction, (4) Students who score high on school satisfaction and/or PE satisfaction are associated with lower risk of reporting symptoms of mental health problems, compared to those who perceive dissatisfaction, and (5) Students with good sleep quality and/or who engage in a healthier lifestyle, either through regular physical activity and/or a healthy diet, experience fewer symptoms of psychological problems, compared to those with low sleep quality and/or have a less healthy lifestyle.

## 2. Materials and Methods

### 2.1. Study Design

A cross-sectional electronic survey was conducted to collect data on students’ perceived symptoms of mental health problems and potential associative demographic characteristics, lifestyle factors, as well as self-rated and school-related wellbeing. Data collections were handled by questionnaires created with nettskjema.no, survey solution developed and hosted by the University of Oslo (nettskjema@usit.uio.no). Electronic survey method was chosen for a more efficient access to the students, to enable geographically dispersed distribution for best possible representativeness and low operating costs.

### 2.2. Recruitment of Participants

Prior to the survey itself, a categorization of all 420 Norwegian upper secondary schools was carried out, with the aim of inviting a proportionately equal number of schools based on the population sizes in the 11 counties, as well as an equal proportion of schools with general study programmes and vocational study programmes, respectively. In this distribution process, we ended up with a total of 265 schools (varying from 18 to 44 in each county), corresponding to approx. 65% of all Norwegian upper secondary schools. A short e-mail invitation was sent with brief information about the study and ethical approval to principals, with a request to provide feedback within 3 weeks on whether the school wanted to participate in the survey or not. Out of a total of 265 schools, the 7 (3%) representing 6 different counties with scattered geographical location agreed to participate: County 1; 2 schools, n = 71 (14%) and n = 29 (6%), respectively, County 2; 1 school, n = 128 (10%), County 3; 1 school, n = 39 (13%), County 4; 1 school, n = 44 (6%), County 5; 1 school, n = 42 (6%) and County 6; 1 school, n = 57 (6%). Out of the remaining schools, 212 (80%) did not respond to the inquiry, 41 (15%) refused to participate, and, finally, 5 (2%) initially said yes but still did not participate.

### 2.3. Survey Development and Validation

As mentioned above, the survey comprised questions covering the following five main topics; (i) symptoms of mental health problems, (ii) demographic characteristics (gender, anthropometric data, socioeconomic status, social contacts/friends and study programme affiliation), (iii) lifestyle factors (sleep problems, leisure physical activity (PA) level and dietary habits), (iv) self-perceived body image, and (v) school satisfaction and satisfaction with PE class participation.

#### 2.3.1. Symptoms of Mental Health Problems

The Hopkins Symptoms Check list (HSCL-25) is a comprehensive, systematized, semi-directed, clinically self-administered questionnaire and the specificity compared with clinical interview is robust, i.e., between 0.78 and 0.88, with an associated reliability (Alpha de Cronbach) between 0.87 and 0.97 [27]. The HSCL-25 consists of the main question: ‘Assess how much each symptom has been a nuisance or inconvenience to you in the last 14 days’, with 25 statements related to mental health and the associated four response categories; ‘not at all’ (1), ‘a little’ (2), ‘quite a bit’ (3), and ‘extremely’ (4). The statements in this questionnaire were as follows: ‘Headaches’, ‘Worrying to much about things’, ‘Feeling tense’, ‘Difficulties falling asleep’, ‘Loss of sexual interest’, ‘Nervousness or shakiness’, ‘Feeling blue’, ‘Crying easily’, ‘Feeling fearful’, ‘Feeling low in energy’, ‘Feeling everything is an effort’, ‘Feeling lonely’, ‘Feeling hopeless about the future’, ‘Heart pounding or racing’, ‘Blaming yourself for things’, ‘Feeling no interest in things’, ‘Faintness, dizziness’, ‘Suddenly scared for no reason’, ‘Restless can’t sit still’, ‘Feelings of worthlessness’, ‘Feelings of being trapped or caught’, ‘Poor appetite’, ‘Trembling’, ‘Spells of terror or panic’, and ‘Thoughts of ending your life’. The average score is calculated by dividing the total sum by 25. An average score of 1.75 or above is an indication of significant mental problems or impaired mental health [28].

#### 2.3.2. Demographic Characteristics

The introductory questions dealt with gender (girl or boy), and the anthropometric data height (cm) and weight (kg), which were converted to body mass index (BMI) according to the following formula: BMI = kg/m^2^ [29].

Social contacts/friends were measured by the question ‘How often do you spend time with good friends? Do not count on members of your own family’ with the following six response categories; ‘I have no good friends’ (1), ‘less often than every year’ (2), ‘sometimes a year’ (3), ‘almost every month, but not weekly’ (4), ‘almost every week, but not daily’ (5), and ‘almost on a daily basis’ (6) [30]. Due to a relatively small number of responses to the two first categories, i.e., ‘I have no good friends’ and ‘less often than every year’, those two categories were merged with category 3 (‘sometimes a year’), implying that a total of four categories were included in the analyses.

Study programme affiliation was measured by the question ‘On which study programme are you affiliated?’ with the 16 following response categories; ‘Sports and Physical Education’ (1), ‘Art, Design and Architecture’ (2), ‘Media and Communication’ (3), ‘Music, Dance and Drama’ (4), ‘Education Programme for Specialization in General Studies’ (5), ‘Building and Constructing’ (6), ‘Electrical and Computer Technology’ (7), ‘Hairdresser, Flowers, Interior and Exposure Design’ (8), ‘Crafts, Design and Product Development’ (9), ‘Health and Youth Development’ (10), ‘Information Technology and Media Communication’ (11), ‘Agriculture, Fishing and Forestry’ (12), ‘Restaurant, Food and Beverages’ (13), ‘Sales, Service and Tourism’ (14), ‘Technology and Industrial’ (15), ‘Supplementary Studies Qualifying for Higher Education’ (16) [31]. Due to the varying number of categories answered, the original study programmes were recoded into two main categories based on criteria of either being (i) Programme for General Studies (n = 285), or (ii) Vocational Studies (n = 125).

#### 2.3.3. Lifestyle Factors

Self-reported sleep problems during the past three months were assessed using the following four single-items derived from a modified version of the Karolinska Sleep Questionnaire [32]: (i) ‘how often did you have problems falling asleep?’, (ii) ‘how often did you wake up too early and could not fall asleep again?’, (iii) ‘how often did you wake up several times and were unable to fall asleep again?’, and (iv) ‘how often was your sleep poor and disturbed?’ [33]. The disturbed sleep score was ranged on 5-point Likert scale comprising the following options: ‘never’ (1), ‘rarely’ (2), ‘sometimes’ (3), ‘very often’ (4), and ‘always’ (5). High scores represent poorer sleep. Based on the Cronbach’s Alpha estimate for the four sub-questions corresponding to α = 0.83, the total score, i.e., the sum of the four subscales divided by 4, was used in the further statistical analyses.

The level of leisure physical activity (PA) was measured by the International Physical Activity Questionnaire (IPAQ short) [34]. The questions include both frequency (number of days per week) and time consumption (hours and minutes) for the following three different intensity levels; walking, moderate, and high. The participants’ energy consumption was calculated based on rest level (equivalent to 1 metabolic equivalent (MET), with the following values used for the three respective intensity levels: walking = 3.3 METs, moderate PA = 4.0 METs and vigorous PA = 8.0 METs. Using these values, four continuous scores are defined: walking MET-minutes/week = 3.3 × walking minutes × walking days moderate MET-minutes/week = 4.0 × moderate-intensity activity minutes × moderate days vigorous MET-minutes/week = 8.0 × vigorous-intensity activity minutes × vigorous-intensity days. Total physical activity MET-min weekly consumption is calculated according to the following formula: MET level × frequency (number of days) × duration (hours and minutes). Based on these estimates, participants are then categorized into the three physical activity categories, low (<600 METs), moderate (600–2999 METs), and high (>3000 METs) [34].

Dietary habits were measured through the following question ‘How would you evaluate you own dietary habits?’ with the following four response categories; ‘unhealthy’ (1), ‘quite healthy’ (2), ‘very healthy’ (3), and ‘I don’t know’ (4) [35]. Due to that the response category ‘I don’t know’ did not represent a meaningful level in the logistic analysis model, those responses were set to ‘missing values’ and excluded from the analyses (n = 12).

#### 2.3.4. Self-Perceived Body Image

Self-perceived body image is a complex construct comprising thoughts, feelings, evaluations, and behaviours related to one’s body [36]. The question was derived from Pisa 2018 Wellbeing questionnaire [37] with the following wording: ‘Thinking about yourself, how much do you agree with each of the following statements?’ ‘I like my look just the way it is’, ‘I consider myself to be attractive’, ‘I am not concerned about my weight’, ‘I like my body’, and ‘I like the way my clothes fit me’. Each of the statements comprised the following five response categories; ‘Strongly disagree’ (1), ‘Disagree’ (2), ‘Agree’ (3), ‘Strongly agree’ (4) and ‘I don’t have an opinion’ (5). Due to that the response category ‘I don’t have an opinion’ did not represent a meaningful value in the calculation of averages, those responses were set to ‘missing values’ (ranging from n = 10 to n = 24 for the respective five statements) and excluded from the analyses. Based on the Cronbach Alpha estimate for the five sub-questions corresponding to α = 0.90, the total score, i.e., the sum of the five subscales divided by 5 was used in the further statistical analyses.

#### 2.3.5. School Satisfaction and Satisfaction with Physical Education (PE) Class Participation

Satisfaction related to school and satisfaction with PE class participation was measured using the two following questions; (i) ‘In general, how do you thrive at school today?’ and (ii) ‘In general, how do you thrive during PE class participation?’. Each of the two questions comprised the following four response categories; ‘poor’ (1), ‘not very good’ (2), ‘good’ (3), and ‘very good’ (4). Due to a relatively low number of responses to the first category ‘poor’ for both questions, this category was merged with the second category ‘not very good’, implying that a total of three categories were included in the analyses.

### 2.4. Statistical Analyses

All statistical analyses were performed using SPSS version 28.0 [38]. To be able to evaluate the associations between the independent predictor variables and the dependent variable we used a binary logistic regression analyses [39]. Prior to the analyses, standard assumption testing, including multicollinearity, outliers, variance, and linearity, were performed and approved. An average score of 1.75 or above was used as an cut-off value and an indication of significant mental problems, or impaired mental health [28], i.e., the following coding was performed; 0 = no (symptoms of mental health problems), and 1 = yes (symptoms of mental health problems). Furthermore, for evaluating any associations between self-perceived body image and school satisfaction and satisfaction with PE class participation, respectively, Spearman’s rank correlation coefficient (rho) [40] was used. The correlation value, which varies between −1 (strong negative correlation) and 1 (strong positive correlation) and with 0 as an indication of no correlation, was further evaluated based on Cohen’s effect sizes; i.e., small correlation (r = 0.10 to 0.29), moderate correlation (r = 0.30 to 0.49), and strong correlation (r = 0.50 to 1.0) [41].

## 3. Results

### 3.1. Demographic Characteristics

Out of the total number of participants in this study (n = 410), the total average age was 17.6 ± 1.4 years, 256 (62.4%) were girls, and the total average body mass index (BMI) was 22.6 ± 3.5 kg/m^2^ (Table 1). The proportion of students at the three different grade levels was as follows; upper secondary level 1 (1st year): n = 171, upper secondary level 2 (2nd year): n = 147, and upper secondary level 3 (3rd year): n = 92.

### 3.2. Associations between the Predictors and Symptoms of Mental Health Problems

A binary logistic regression analyses was performed to assess the impact of a number of factors on the likelihood that the students would report that they had symptoms of mental health problems. The model comprised nine independent variables (gender, self-perceived body image, sleep problems, leisure physical activity level, dietary habits, social contacts/friends, study programme affiliation, school satisfaction, and satisfaction with PE class participation). The full model containing all predictors was statistically significant χ^2^ (15, n = 340) = 186.40, *p* < 0.001, indicating that the model was able to distinguish between students who reported and did not report a mental health problem. The model as a whole explained between 42% (Cox & Snell R square) and 56% (Nagelkerke R square) of the variance in symptoms of mental health problems, and correctly classified 80% of cases. As shown in Table 2, six of the independent variables made a unique statistically significant contribution to the model (gender, self-perceived body image, sleep problems, dietary habits, school satisfaction, and satisfaction with PE class participation). The strongest predictor of reporting symptoms of mental health problems was gender, i.e., being a girl (OR = 4.15, 95% CI 2.22, 7.76, *p* < 0.001). This indicate that girls were four times more likely to report having symptoms of mental health problems (HSCL-25 score > 1.75), compared to the boys when all other factors in the model are controlled for. The second strongest predictor was sleep problems (OR = 2.92, 95% CI 1.91, 4.48, *p* < 0.001), indicating that one unit increase in perceived sleep problems (on a 5-point scale) lead to an almost three times higher likelihood of reporting symptoms of mental health problems. The next significant predictor was self-perceived body image (OR = 0.41, 95% CI 0.26, 0.65, *p* < 0.001), indicating that for every unit increase in self-perceived body image (on a 4-point scale), the students were 0.41 times (59%) less likely to report having symptoms of mental health problems. The students’ dietary habits did also have a significant impact on the dependent variable, i.e., those reporting having a quite healthy diet were 0.42 times (58%) less likely to report having symptoms of mental health problems than those reporting unhealthy dietary habits (OR = 0.42, 95% CI 0.19, 0.93, *p* = 0.033). Furthermore, the analyses revealed that both students’ school satisfaction (OR = 0.18, 95% CI 0.05, 0.70, *p* =0.013), as well as satisfaction with PE class participation (OR = 0.32, 95% CI 0.12, 0.87, *p* = 0.025), had a significant impact on the likelihood of reporting symptoms of mental health problems. This implies that students who reported the highest satisfaction level (very good) on the questions related to school satisfaction and satisfaction with PE class participation, respectively, were 82% and 68% less likely to report having symptoms of mental health problems compared to those reporting the lowest level of satisfaction (poor/not very good).

Based on our main statistical model, neither study programme affiliation, leisure PA level, nor social contacts/friends showed any statistically significant association with the symptoms of mental health problems (Table 2).

Based on Spearman’s correlation analyses positive relationships were observed between self-perceived body image and school satisfaction (r (400) = 0.24, *p* < 0.001) and satisfaction with PE class participation (r (400) = 0.30, *p* < 0.001), respectively. In addition, a Spearman’s correlation analyses were performed between the original dependent variable (i.e., symptoms of mental health problems on a ratio scale) and self-perceived body image (r (400) = −0.53, *p* < 0.001).

## 4. Discussion

This cross-sectional survey among upper secondary school students aimed to evaluate potential associations between different adolescent characteristics and symptoms of mental health problems. The results indicate that both gender, self-perceived body image, sleep, dietary habits, school satisfaction and students’ satisfaction with PE class participation are significant predictors of young people’s mental health.

With regards to hypothesis 1, being a girl was associated with a significantly higher prediction for reporting symptoms of mental health problems compared to boys, which is consistent with a previous meta-analysis by Nolen-Hoeksma and Girgus (2019), suggesting that girls aged 15 years and older were twice as likely to experience depression compared to boys [42]. The reasons for gender differences in mental health problems among adolescents are not fully understood, and previous research has pointed to both biological gender differences [43,44], as well as being related to gender perceptions and the socially defined role of girls and boys, which in many societies exposes them to gender-specific stress factors [45]. Furthermore, it has been suggested that boys acknowledge their mental health problems to a lesser extent than girls (who report more internalizing disorders, such as depression and anxiety) and tend to mask their mental health problems through acting out behaviour, which can result in externalized difficulties, such as antisocial personality disorders, substance abuse, and/or addiction [45]. In the context of gender differences, previous research has also indicated that loneliness is more strongly associated with increased symptoms of depression in girls [46], whereas it is associated with increased social anxiety in boys [47]. Although our main statistical model did not reveal any significant associations between social contacts/friends and symptoms of mental health problems (our hypothesis 2), the post-hoc gender difference analyses revealed a tendency for an impact in boys whereby those who reported daily contact with friends were 0.12 times (88%) less likely to report symptoms of mental health problems compared to those who spent time with friends only a few times a year or never (*p* = 0.08).

With regards to our hypothesis 3, the logistic regression model, including both boys and girls, revealed significant associations between self-perceived body image and symptoms of mental health problems, which is in accordance with previous studies [48,49]. However, additional post-hoc gender difference analyses revealed that this association only remained significant for the girls (*p* < 0.001), whereas only a tendency was observed for the boys (*p* = 0.065). In this context, it may be relevant to refer to a previous meta-analysis by Groesz et al., suggesting that exposure to obvious thin media images leads to a decrease in body satisfaction among girls under 19 years of age [50]. Furthermore, it is argued that today’s diet, fitness, and beauty trends shown in reality TV programs and social media can contribute to unhealthy body perceptions in young people [51]. In this regard, our study suggests a future focus on preventing body stigma in school, especially among girls, as well as spreading knowledge about positive health choices. In addition, researchers should seek to make use of methodological approaches aimed at succeeding with lifestyle changes related to both diet and physical activity, as well as healthy social media habits [49].

With regards to hypothesis 4, our analyses showed that those who experience a high degree of school satisfaction and satisfaction with PE class participation, respectively, were associated with significantly lower odds of having symptoms of mental health problems, compared to those who experience a high degree of dissatisfaction. However, in girls, the post-hoc gender difference analyses revealed no significant impact of neither school satisfaction, nor satisfaction with PE class participation on symptoms of mental health problems, whereas those associations increased in boys. Although our observations are not directly comparable to previous studies, it may be of relevance to point out that previous research on motivation for PE have suggested that boys tend to have higher motivation/satisfaction with PE class participation compared to girls. For instance, by way of boys receiving more positive feedback than girls, and boys being more likely to choose their preferred physical activities compared to girls. [52,53]. In terms of school satisfaction, a large international time trend study among 15-year-old students carried out between 2002–2018 showed a flattening of the gender differences recorded in the early 2000s (i.e., in a period when more girls liked school a lot than boys) as a result of more boys liking school a lot [22]. In contrast, the latter study revealed an increase in perceived school pressure among girls, which is in line with research showing that school burnout is more prevalent among girls compared to boys [54]. This emphasizes the importance of the teachers knowing their responsibilities and having the ability to facilitate teaching and provide feedback in such a way that it is perceived as meaningful and motivating for the students regardless of gender.

Although our findings are supported by previous research that suggest a positive association between school satisfaction and sound mental health [55,56,57], only a limited number of studies have evaluated the association between perceived satisfaction with PE class participation and mental health [58]. In this context, a previous meta-analysis that focused only on PE participation (not satisfaction), found no evidence for a relationship between PE participation and mental health [59]. However, given the significant association between symptoms of mental health problems and self-perceived body image [60], which was also derived from our main statistical model (Table 2) and supported by a strong negative correlation (r (400) = −0.53, *p* < 0.001), it may be appropriate to include that according to a previous structured review, it has been suggested that PE may play a potentially beneficial role in future body image school-based interventions [61], which seems reasonable considering that previous research has shown a positive relationship between school satisfaction and self-perceived body image [62,63]. Such a relationship was otherwise supported by our study through Spearman’s correlation analyses that showed moderate positive relationships between self-perceived body image and both school satisfaction (r = 0.41, *p* < 0.001) and satisfaction with PE class participation (r = 0.31, *p* < 0.001), respectively. This underscores the importance of encouraging PE teachers to promote a healthy body image among young people through motor and sports activity programs and planning social guidelines to improve students’ self-perceived body image [64].

Finally, with regards to hypothesis 5, our study showed a significant association between a higher prediction of reporting symptoms of mental health problems in students who have experienced sleep problems, which is consistent with previous research [65,66]. Moreover, it is suggested that there is a bidirectional relationship between the two conditions, in which either can be a cause or result of the other [67], which, based on our study design, is difficult to identify, however. Furthermore, the excessive use of social media has been suggested as a mediator for lower sleep quality in adolescence [68]. Although we lack information to support this relationship, this is also probably a contributing factor in our target group. Given that sleep quality has been shown to be important in terms of students’ mental health, but also their cognitive capacity and school performance [66,69,70], our finding is nevertheless an important confirmation of the importance of increasing students’ self-awareness around sufficient sleep to prevent mental health problems and maximize their school performance.

In comparison to those perceiving their dietary habits as unhealthy, students in the category ‘quite healthy’ were 58% less likely of reporting symptoms of mental health problems, which is in accordance with previous research suggesting such a relationship [20,71]. The association between symptoms of mental health problems and dietary patterns may be the result of several potential mechanisms, including biological links and psychosocial explanations [72]. However, as unhealthy eating is associated with the maintenance of a pro-inflammatory state [73], inflammation has been proposed as a leading theory to link diet and mental health symptoms, especially depressive symptoms [74].

Our study indicated no associations between leisure PA level and symptoms of mental health problems, which was somewhat surprising given that previous meta-analyses and structured reviews have suggested that physical activity may be beneficial for children and adolescents’ mental health, while sedentary time on the contrary has been associated with increased risk for mental health problems [75,76]. A potential explanation for the lack of impact of leisure PA level on symptoms of mental health problems in our binary logistic regression model may be related to the fact that the study was conducted shortly after the reopening of society following the COVID-19 pandemic, i.e., a period of documented decrease in physical activity among children and adolescents [77], however, it is important to emphasize that this is only based on speculation.

### Limitations

The limitations of the study are the use of cross-sectional design that do not allow us to establish any cause-and-effect relationship, as well as the use of self-reported data. Furthermore, the use of single-item measures for some of the predictor variables constitutes a potential limitation as these questions provide limited knowledge about the reliability of the measurements. An additional weakness is our limited knowledge of the invited schools and the characteristics of the non-participating students. Finally, it should be mentioned that the relatively modest response rate could potentially affect the representativeness of the study.

## 5. Conclusions

This study among Norwegian upper secondary school students confirms that mental health problems are significantly more prevalent among girls than among boys. Furthermore, the results indicate a significant association between the symptoms of mental problems, sleep problems, and dietary habits, as well as in relation to dissatisfaction with body image, school life in general, and physical education class participation.

## Figures and Tables

**Table 1:** Descriptive statistics by gender for age, anthropometric data, average score on the dependent variable ‘symptoms of mental health problems’ (HSCL-25) (measured on a ratio scale), as well as average score for the predictor variables measured at either ratio or ordinal level. The *p*-values refer to the gender differences.

Characteristics	Boys (n = 154)		Girls (n = 256)		Total (n = 410)		*p*-Value
	Mean	SD	Mean	SD	Mean	SD	
Age (years)	17.7	1.5	17.5	1.4	17.6	1.4	0.468
Height (cm)	180.4	7.0	166.6	6.2	171.8	9.3	<0.001
Weight (kg)	73.7	13.9	63.0	10.5	67.1	13.0	<0.001
BMI (kg/m^2^)	22.6	3.6	22.7	3.3	22.6	3.5	0.797
HSCL-25 (1–4)	1.51	0.46	2.04	0.63	1.84	0.62	<0.001
Body image (1–4)	2.91	0.69	2.50	0.77	2.65	0.77	<0.001
Sleep problems (1–5)	2.26	0.81	2.54	0.86	2.43	0.85	0.002
Leisure PA level (1–3)	2.08	0.77	2.01	0.67	2.04	0.71	0.360
Dietary habits (1–3)	1.86	0.55	1.80	0.48	1.82	0.51	0.249
School satisfaction (1–3)	2.30	0.57	2.16	0.64	2.21	0.62	0.033
Satisfaction PE (1–3)	2.46	0.64	2.13	0.70	2.25	0.70	<0.001
Social contacts/friends (1–4)	3.03	0.91	2.85	0.81	2.92	0.85	0.045

**Table 2:** Results from the binary logistic regression analyses.

Predictor Variables							95% CI for Exp(B)	
	B	S.E.	Wald	Df	Sig.	Exp(B)	Lower	Upper
Gender	1.423	0.319	19.897	1	<0.001	4.151	2.221	7.760
Study programme affiliation	−0.454	0.339	1.797	1	0.180	0.635	0.327	1.234
Body image (1–4)	−0.897	0.236	14.389	1	<0.001	0.408	0.257	0.648
Sleep problems (1–5)	1.072	0.218	24.242	1	<0.001	2.921	1.907	4.477
Leisure PA level			0.310	2	0.857			
Leisure PA level (1)	0.174	0.428	0.166	1	0.683	1.191	0.515	2.753
Leisure PA level (2)	0.003	0.490	0.000	1	0.995	1.003	0.384	2.623
Dietary habits			4.652	2	0.098			
Dietary habits (1)	−0.871	0.408	4.566	1	0.033	0.418	0.188	0.930
Dietary habits (2)	−0.554	0.819	0.457	1	0.499	0.575	0.115	2.861
School satisfaction			7.022	2	0.030			
School satisfaction (1)	−1.028	0.630	2.663	1	0.103	0.358	0.104	1.229
School satisfaction (2)	−1.692	0.682	6.153	1	0.013	0.184	0.048	0.701
Satisfaction with PE participation			5.711	2	0.058			
Satisfaction PE (1)	−0.593	0.500	1.410	1	0.235	0.553	0.207	1.471
Satisfaction PE (2)	−1.145	0.512	5.009	1	0.025	0.318	0.117	0.867
Social contacts/friends			2.501	3	0.321			
Social contacts/friends (1)	0.317	0.746	0.181	1	0.670	1.374	0.318	5.925
Social contacts/friends (2)	−0.084	0.672	0.016	1	0.900	0.919	0.246	3.432
Social contacts/friends (3)	−0.584	0.714	0.669	1	0.414	0.558	0.138	2.262
Constant	1.606	1.135	2.003	1	0.157	4.985		

## Data Availability

The datasets generated during and/or analysed during the current study are available from the corresponding author on reasonable request.

## References

[1] World Health Organization (2004). Promoting Mental Health: Concepts, Emerging Evidence, Practice: Summary Report.

[2] Eddolls W.T.B., McNarry M.A., Lester L., Winn C.O.N., Stratton G., Mackintosh K.A. (2018). The association between physical activity, fitness and body mass index on mental well-being and quality of life in adolescents. Qual. Life Res..

[3] Patel V., Flisher A.J., Hetrick S., McGorry P. (2007). Mental health of young people: A global public-health challenge. Lancet.

[4] Moksnes U.K., Reidunsdatter R.J. (2019). Self-esteem and mental health in adolescents–level and stability during a school year. Nor. Epidemiol..

[5] World Health Organization Adolscent Mental Health. https://www.who.int/news-room/fact-sheets/detail/adolescent-mental-health.

[6] World Health Organization (2019). The WHO Special Initiative for Mental Health (2019–2023): Universal Health Coverage for Mental Health.

[7] Collishaw S. (2015). Annual research review: Secular trends in child and adolescent mental health. J. Child Psychol. Psychiatry.

[8] de Oliveira J.M.D., Butini L., Pauletto P., Lehmkuhl K.M., Stefani C.M., Bolan M., Guerra E., Dick B., De Luca Canto G., Massignan C. (2022). Mental health effects prevalence in children and adolescents during the COVID-19 pandemic: A systematic review. Worldviews Evid. Based Nurs..

[9] Viner R., Russell S., Saulle R., Croker H., Stansfield C., Packer J., Nicholls D., Goddings A.-L., Bonell C., Hudson L. (2022). School Closures During Social Lockdown and Mental Health, Health Behaviors, and Well-being Among Children and Adolescents During the First COVID-19 Wave: A Systematic Review. JAMA Pediatr..

[10] Aldridge J.M., McChesney K. (2018). The relationships between school climate and adolescent mental health and wellbeing: A systematic literature review. Int. J. Educ. Res..

[11] Stansfeld S., Clark C., Bebbington P., King M., Jenkins R., Hinchliffe S. (2016). Common Mental Disorders.

[12] Keles B., McCrae N., Grealish A. (2020). A systematic review: The influence of social media on depression, anxiety and psychological distress in adolescents. Int. J. Adolesc. Youth.

[13] Pullmer R., Chung J., Samson L., Balanji S., Zaitsoff S. (2019). A systematic review of the relation between self-compassion and depressive symptoms in adolescents. J. Adolesc..

[14] Reiss F. (2013). Socioeconomic inequalities and mental health problems in children and adolescents: A systematic review. Soc. Sci. Med..

[15] Mannan M., Mamun A., Doi S., Clavarino A. (2016). Prospective associations between depression and obesity for adolescent males and females—A systematic review and meta-analysis of longitudinal studies. PLoS ONE.

[16] Kaur G. (2018). Mental health of adolescents in school settings: A review. Indian J. Health Wellbeing.

[17] Mammen G., Faulkner G. (2013). Physical activity and the prevention of depression: A systematic review of prospective studies. Am. J. Prev. Med..

[18] Rodriguez-Ayllon M., Cadenas-Sánchez C., Estévez-López F., Muñoz N.E., Mora-Gonzalez J., Migueles J.H., Molina-García P., Henriksson H., Mena-Molina A., Martínez-Vizcaíno V. (2019). Role of Physical Activity and Sedentary Behavior in the Mental Health of Preschoolers, Children and Adolescents: A Systematic Review and Meta-Analysis. Sports Med..

[19] Kulkarni A.A., Swinburn B.A., Utter J. (2015). Associations between diet quality and mental health in socially disadvantaged New Zealand adolescents. Eur. J. Clin. Nutr..

[20] O’Neil A., Quirk S.E., Housden S., Brennan S.L., Williams L.J., Pasco J.A., Berk M., Jacka F.N. (2014). Relationship Between Diet and Mental Health in Children and Adolescents: A Systematic Review. Am. J. Public Health.

[21] Esmaeelzadeh S., Moraros J., Thorpe L., Bird Y. (2018). Examining the Association and Directionality between Mental Health Disorders and Substance Use among Adolescents and Young Adults in the U.S. and Canada—A Systematic Review and Meta-Analysis. J. Clin. Med..

[22] Löfstedt P., García-Moya I., Corell M., Paniagua C., Samdal O., Välimaa R., Lyyra N., Currie D., Rasmussen M. (2020). School Satisfaction and School Pressure in the WHO European Region and North America: An Analysis of Time Trends (2002–2018) and Patterns of Co-occurrence in 32 Countries. J. Adolesc. Health.

[23] Mulloy M., Weist M. (2013). Implementing a Public Mental Health Framework Within Schools.

[24] Liu J., Bartholomew K., Chung P.-K. (2017). Perceptions of teachers’ interpersonal styles and well-being and ill-being in secondary school physical education students: The role of need satisfaction and need frustration. Sch. Ment. Health.

[25] The Education Act (1998). Act Relating to Primary and Secondary Education and Training (LOV-1998-07-17-61).

[26] The Norwegian Directorate for Education and Training (2018). Fagfornyelsen—Overordnet del (The National Norwegian K-12 Curriculum).

[27] Nabbe P., Le Reste J.Y., Guillou-Landreat M., Gatineau F., Le Floch B., Montier T., Van Marwijk H., Van Royen P. (2019). The French version of the HSCL-25 has now been validated for use in primary care. PLoS ONE.

[28] Sandanger I., Moum T., Ingebrigtsen G., Dalgard O.S., Sørensen T., Bruusgaard D. (1998). Concordance between symptom screening and diagnostic procedure: The Hopkins Symptom Checklist-25 and the Composite International Diagnostic Interview I. Soc. Psychiatry Psychiatr. Epidemiol..

[29] Keys A., Fidanza F., Karvonen M.J., Kimura N., Taylor H.L. (1972). Indices of relative weight and obesity. J. Chronic Dis..

[30] The Norwegian Directorate of Health (2018). Quality of Life—Recommendations for a Better Measurement System.

[31] Utdanningsdirektoratet Utdanningsløpet Videregående Opplæring. https://www.udir.no/utdanningslopet/videregaende-opplaring/.

[32] Åkerstedt T., Hume K., Minors D., Waterhouse J. (1994). The meaning of good sleep: A longitudinal study of polysomnography and subjective sleep quality. J. Sleep Res..

[33] Hansen Å.M., Gullander M., Hogh A., Persson R., Kolstad H.A., Willert M.V., Bonde J.P., Kaerlev L., Rugulies R., Grynderup M.B. (2016). Workplace bullying, sleep problems and leisure-time physical activity: A prospective cohort study. Scand. J. Work. Environ. Health.

[34] Craig C.L., Marshall A.L., Sjöström M., Bauman A.E., Booth M.L., Ainsworth B.E., Pratt M., Ekelund U., Yngve A., Sallis J.F. (2003). International physical activity questionnaire: 12-country reliability and validity. Med. Sci. Sports Exerc..

[35] The Norwegian Directorate of Health (2002). Ungkost—2000 Nationally Representative Dietary Survey in Norwegian 4th and 8th Graders.

[36] Hosseini S.A., Padhy R.K. (2019). Body Image Distortion.

[37] OECD PISA 2018 Well-Being Framework. https://www.oecd-ilibrary.org/content/component/38a34353-en.

[38] IBM Corp. (2021). Released 2021. IBM SPSS Statistics for Windows, Version 28.0.

[39] Tabachnick B.G., Fidell L.S., Ullman J.B. (2007). Using Multivariate Statistics.

[40] Watson R., Atkinson I., Egerton P. (2017). Successful Statistics for Nursing and Healthcare.

[41] Pallant J. (2013). SPSS survival manual. A Step by Step Guide to Data Analysis.

[42] Nolen-Hoeksema S., Girgus J.S. (1994). The emergence of gender differences in depression during adolescence. Psychol. Bull..

[43] Eid R.S., Gobinath A.R., Galea L.A.M. (2019). Sex differences in depression: Insights from clinical and preclinical studies. Prog. Neurobiol..

[44] Soares C.N. (2014). Mood disorders in midlife women: Understanding the critical window and its clinical implications. Menopause.

[45] Van Droogenbroeck F., Spruyt B., Keppens G. (2018). Gender differences in mental health problems among adolescents and the role of social support: Results from the Belgian health interview surveys 2008 and 2013. BMC Psychiatry.

[46] Liu H., Zhang M., Yang Q., Yu B. (2020). Gender differences in the influence of social isolation and loneliness on depressive symptoms in college students: A longitudinal study. Soc. Psychiatry Psychiatr. Epidemiol..

[47] Mak H.W., Fosco G.M., Feinberg M.E. (2018). The role of family for youth friendships: Examining a social anxiety mechanism. J. Youth Adolesc..

[48] Taylor V.S., Ye J., Mack D., Fry-Johnson Y., Smith Q., Harris C.L. (2011). Overweight in School-Aged Children Associated With Emotional and Behavioral Difficulties: Results From a National Sample. J. Natl. Med. Assoc..

[49] Gillen M.M. (2015). Associations between positive body image and indicators of men’s and women’s mental and physical health. Body Image.

[50] Groesz L.M., Levine M.P., Murnen S.K. (2002). The effect of experimental presentation of thin media images on body satisfaction: A meta-analytic review. Int. J. Eat. Disord..

[51] Voelker D.K., Reel J.J., Greenleaf C. (2015). Weight status and body image perceptions in adolescents: Current perspectives. Adolesc. Health Med. Ther..

[52] Granero-Gallegos A., Baena-Extremera A., Pérez-Quero F.J., Ortiz-Camacho M.M., Bracho-Amador C. (2012). Analysis of motivational profiles of satisfaction and importance of physical education in high school adolescents. J. Sports Sci. Med..

[53] Lemes V.B., Araujo Gaya A.C., Brand C., Dias A.F., Cristi-Montero C., Mota J., Gaya A.R. (2021). Associations among psychological satisfaction in physical education, sports practice, and health indicators with physical activity: Direct and indirect ways in a structural equation model proposal. Int. J. Pediatr. Adolesc. Med..

[54] Salmela-Aro K., Kiuru N., Pietikäinen M., Jokela J. (2008). Does school matter? The role of school context in adolescents’ school-related burnout. Eur. Psychol..

[55] Baker J.A., Dilly L.J., Aupperlee J.L., Patil S.A. (2003). The developmental context of school satisfaction: Schools as psychologically healthy environments. Sch. Psychol. Q..

[56] Baker J.A., Maupin A.N. (2009). School satisfaction and children’s positive school adjustment. Handbook of Positive Psychology in Schools.

[57] Cheon H., Lim S. (2020). Pursuing Sustainable Happiness through Participation in Exercise for South Korean Students: Structural Relationships among Exercise, Mental Health Factors, School Satisfaction, and Happiness. Sustainability.

[58] Standage M., Gillison F. (2007). Students’ motivational responses toward school physical education and their relationship to general self-esteem and health-related quality of life. Psychol. Sport Exerc..

[59] White R.L., Babic M.J., Parker P.D., Lubans D.R., Astell-Burt T., Lonsdale C. (2017). Domain-Specific Physical Activity and Mental Health: A Meta-analysis. Am. J. Prev. Med..

[60] Tiwari G.K., Kumar S. (2015). Psychology and body image: A review. Shodh Prerak.

[61] Kerner C., Haerens L., Kirk D. (2017). Understanding body image in physical education: Current knowledge and future directions. Eur. Phys. Educ. Rev..

[62] Reschly A.L., Huebner E.S., Appleton J.J., Antaramian S. (2008). Engagement as flourishing: The contribution of positive emotions and coping to adolescents’ engagement at school and with learning. Psychol. Sch..

[63] Raufelder D., Waak S., Melchior A., Ittel A. (2013). The role of sport involvement and general self-worth in the interplay between body dissatisfaction, worry, and school disaffection in preadolescent boys and girls. Child Dev. Res..

[64] Fischetti F., Latino F., Cataldi S., Greco G. (2020). Gender differences in body image dissatisfaction: The role of physical education and sport. J. Hum. Sport Exerc.

[65] Scott A.J., Webb T.L., Martyn-St James M., Rowse G., Weich S. (2021). Improving sleep quality leads to better mental health: A meta-analysis of randomised controlled trials. Sleep Med. Rev..

[66] Kansagra S. (2020). Sleep Disorders in Adolescents. Pediatrics.

[67] Kaneita Y., Yokoyama E., Harano S., Tamaki T., Suzuki H., Munezawa T., Nakajima H., Asai T., Ohida T. (2009). Associations between sleep disturbance and mental health status: A longitudinal study of Japanese junior high school students. Sleep Med..

[68] Hale L., Li X., Hartstein L.E., LeBourgeois M.K. (2019). Media Use and Sleep in Teenagers: What Do We Know?. Curr. Sleep Med..

[69] Tarokh L., Saletin J.M., Carskadon M.A. (2016). Sleep in adolescence: Physiology, cognition and mental health. Neurosci. Biobehav. Rev..

[70] Musshafen L.A., Tyrone R.S., Abdelaziz A., Sims-Gomillia C.E., Pongetti L.S., Teng F., Fletcher L.M., Reneker J.C. (2021). Associations between sleep and academic performance in US adolescents: A systematic review and meta-analysis. Sleep Med..

[71] Khalid S., Williams C.M., Reynolds S.A. (2016). Is there an association between diet and depression in children and adolescents? A systematic review. Br. J. Nutr..

[72] Orlando L., Savel K.A., Madigan S., Colasanto M., Korczak D.J. (2022). Dietary patterns and internalizing symptoms in children and adolescents: A meta-analysis. Aust. N. Z. J. Psychiatry.

[73] Perez-Cornago A., de la Iglesia R., Lopez-Legarrea P., Abete I., Navas-Carretero S., Lacunza C.I., Lahortiga F., Martinez-Gonzalez M.A., Martinez J.A., Zulet M. (2014). A decline in inflammation is associated with less depressive symptoms after a dietary intervention in metabolic syndrome patients: A longitudinal study. Nutr. J..

[74] Wang J., Zhou Y., Chen K., Jing Y., He J., Sun H., Hu X. (2019). Dietary inflammatory index and depression: A meta-analysis. Public Health Nutr..

[75] Biddle S.J.H., Ciaccioni S., Thomas G., Vergeer I. (2019). Physical activity and mental health in children and adolescents: An updated review of reviews and an analysis of causality. Psychol. Sport Exerc..

[76] Biddle S.J.H., Asare M. (2011). Physical activity and mental health in children and adolescents: A review of reviews. Br. J. Sports Med..

[77] Rossi L., Behme N., Breuer C. (2021). Physical Activity of Children and Adolescents during the COVID-19 Pandemic—A Scoping Review. Int. J. Environ. Res. Public Health.

